# Tumour‐derived exosome SNHG17 induced by oestrogen contributes to ovarian cancer progression via the CCL13–CCR2–M2 macrophage axis

**DOI:** 10.1111/jcmm.18315

**Published:** 2024-04-28

**Authors:** Haiyan Liang, Shuo Geng, Yadong Wang, Qing Fang, Yongfeng Xin, Yanqing Li

**Affiliations:** ^1^ Department of Obstetrics and Gynecology China‐Japan Friendship Hospital Beijing China; ^2^ Scientific Research Department GeneX Health Co., Ltd Beijing China; ^3^ Institute of Clinical Medicine China‐Japan Friendship Hospital Beijing China; ^4^ Department of Gynecology The People's Hospital of DaLaTe Ordos Inner Mongolia China; ^5^ Department of Gynecology Hebei Provincial Hospital of Traditional Chinese Medicine Wuhan Hebei China

**Keywords:** exosomal SNHG17, M2 macrophage, oestrogen, ovarian cancer

## Abstract

Oestrogen is known to be strongly associated with ovarian cancer. There was much work to show the importance of lncRNA SNHG17 in ovarian cancer. However, no study has revealed the molecular regulatory mechanism and functional effects between oestrogen and SNHG17 in the development and metastasis of ovarian cancer. In this study, we found that SNHG17 expression was significantly increased in ovarian cancer and positively correlated with oestrogen treatment. Oestrogen could promote M2 macrophage polarization as well as ovarian cancer cells SKOV3 and ES2 cell exosomal SNHG17 expression. When exposure to oestrogen, exosomal SNHG17 promoted ovarian cancer cell proliferation, migration, invasion and epithelial‐mesenchymal transition (EMT) in vitro, and tumour growth and lung metastasis in vivo by accelerating M2‐like phenotype of macrophages. Mechanically, exosomal SNHG17 could facilitate the release of CCL13 from M2 macrophage via the PI3K‐Akt signalling pathway. Moreover, CCL13‐CCR2 axis was identified to be involved in ovarian cancer tumour behaviours driven by oestrogen. There results demonstrate a novel mechanism that exosomal SNHG17 exerts an oncogenic effect on ovarian cancer via the CCL13–CCR2–M2 macrophage axis upon oestrogen treatment, of which SNHG17 may be a potential biomarker and therapeutic target for ovarian cancer responded to oestrogen.

## 1 INTRODUCTION

Ovarian cancer is one of the most common malignant tumours with high incidence and mortality rate in women worldwide. Lack of obvious symptoms at early stage and no effective or sensitive clinical screening methods led to ovarian cancer difficult to be diagnosed.^1^, ^2^, ^3^ Studies have found that oestrogen receptor (ER) plays an important role in the growth and metastasis of ovarian cancer, which is expressed in 40% to 80% of ovarian cancer cells and could promote ovarian cancer cells proliferation.^4^ Genetic research in 2021 also revealed that high levels of ER are associated with an increased risk of ovarian cancer.^5^ Although much work has been explored on the mechanism of oestrogen promoting ovarian cancer tumorigenesis, but the existing research results which could reveal this abstruse mechanism remains elusive.

Nowadays, tumour microenvironment (TME) has received increasing attentions on tumour development and metastasis.^6^, ^7^, ^8^ Macrophages can be classified by their activation state as M1 macrophages (classically activated macrophages) or M2 macrophages (alternatively activated macrophages). M1 macrophages could produce inflammatory cytokines such as interleukin (IL)‐6 and IL‐1β, killing tumour cells and pathogenic microorganisms whereas M2 macrophages secret growth factors such as epidermal growth factor (EGF) participating in the tissue remodelling or tumour progression.^9^, ^10^ Previous research showed that tumour‐recruited M2 macrophages promote gastric and breast cancer metastasis via M2 macrophage‐secreted CHI3L1 protein.^11^ A study reported that M2 macrophage‐secreted EGF may suppress LIMT expression via activating EGFR‐ERK signalling pathway to promote ovarian cancer progression.^12^ Increased M2/M1 macrophage ratio induced by RACK1 was found to promote oral squamous cell carcinoma development.^13^ These studies suggest that tumour‐derived M2 macrophages are closely related to tumorigenesis.

Long noncoding RNA (lncRNAs) have been studied to be widely involved in physiological and pathological processes including cancers, autoimmune and cardiac diseases.^14^ Small nucleolar RNA host gene 17 (SNHG17) is a newly discovered tumour‐related lncRNA of the SNHG family which is highly expressed and may exert cancer‐promoting effects in multiple cancers. For example, a present study revealed that SNHG17 could aggravate prostate cancer progression through regulating its homologue SNORA71B via a positive feedback loop.^15^ A previous study showed that SNHG17 promoted gastric cancer progression by epigenetically silencing of p15 and p57.^16^ One study reported that SNHG17 functioned as an oncogenic lncRNA in rectal cancer by regulating the miR‐361‐3p/STC2 axis.^17^ Another report showed that METTL3‐induced SNHG17 could promote lung adenocarcinoma gefitinib resistance by epigenetically repressing LATS2 expression.^18^ These studies indicate that SNHG17 is a crucial regulator for carcinogenesis in human cancers. In addition, the role of SNHG17 has also been investigated in ovarian cancer. SNHG17 was found to promote ovarian cancer cell proliferation and invasion by increasing FOXA1.^19^ A report suggested that SNHG17 acted as an oncogene in ovarian cancer by regulating CDK6.^20^ SNHG17 downregulation inhibited the tumorigenesis of epithelial ovarian cancer via the regulation of miR‐485‐5p/AKT1 axis.^21^ These studies reveal that lncRNA SNHG17 also plays a crucial role in ovarian cancer. However, the detailed function and mechanisms remain largely unknown.

Exosomes secreted by cells act as a key role in mediating cell–cell communication. Emerging evidence suggests that exosomes play an important role in facilitating tumorigenesis in the tumour microenvironment in multiple tumours, of which the aberrant expression of exosomal constituents such as lncRNAs are crucial.^21^, ^22^, ^23^ For instance, it was reported that exosomal lncRNA RPPH1 promoted colorectal cancer metastasis by mediating macrophage M2 polarization.^24^ Exosomal LNMAT2 was found to promote lymphatic metastasis in bladder cancer.^25^ Exosomal metastasis‐associated lung adenocarcinoma transcript 1 (MALAT1) was verified to promote angiogenesis in epithelial ovarian cancer.^26^ In addition, some studies suggest that oestrogen may be associated with the regulation of exosomes release in some human cancers. One research showed that the regulation of oestrogen signalling in breast cancer cells may be related to exosome‐delivered metastasis‐associated protein 1 (MTA1).^27^ Another research showed that exosomes were involved in the transfer of cancer cell resistance to anti‐oestrogen drugs.^28^ One study showed that oestrogen could increase the release of H19‐carrying exosomes from cholangiocytes.^29^ Another study revealed that exosomal miRNA played a critical role in the horizontal transfer of hormonal resistance.^30^ These above findings suggest that the oestrogen is closely related to exosomes and the contents of exosomes such as miRNAs or lncRNAs or others and thus prompted us to study the molecular mechanisms of exosome‐delivered lncRNAs in regulating oestrogen‐induced ovarian cancer tumorigenesis.

In the present study, we hypothesized that oestrogen‐induced exosomes might promote ovarian cancer tumorigenesis by mediating macrophage M2 polarization. We verified that SNHG17 was increased in oestrogen‐induced exosomes. Upon oestrogen treatment, exosome‐delivered SNHG17 promoted macrophage M2 polarization via PI3K/Akt signalling pathway. Furthermore, CCL13‐CCR2 axis was identified to be involved in the regulation of exosomal SNHG17 on promoting oestrogen‐triggered ovarian cancer tumour growth and metastasis in vitro and in vivo. Thus, our work demonstrates a novel mechanism of oestrogen‐induced exosomes on ovarian cancer tumorigenesis.

## 2 METHODS AND MATERIALS

### 2.1 Data processing

The dataset involved (GSE119054) were downloaded from the public Gene Expression Omnibus (GEO) database (https://www.ncbi.nlm.nih.gov/gds/). The dataset GSE119054 sequencing information in the NCBI GEO database is based on GPL19615 (Agilent‐067406 CBC lncRNA + mRNA microarray V4.0) microarray platform, which contains three normal tissue samples and six malignant ovarian tissue samples.^31^ The false discovery rate (FDR) < 0.05 and the absolute value of expression difference fold change (|log2FC|) >= 1.5 were used as thresholds for significant differences, and differential analysis between tumour and control group comparisons was performed using the limma of the R/Bioconductor package and the analysis results were visualized with the ggplot2 and heatmap packages.

### 2.2 Cell culture

Ovarian cancer cell lines SKOV3 and ES2 were bought from Cell Culture Center, Chinese Academy of Medical Sciences (Beijing, China). The characteristics of cell lines were identified. All experiments were implemented with cells without mycoplasma. Cells were cultured using RPMI 1640 (Invitrogen) with 10% foetal calf plasma (Hyclone, Logan, UT), streptomycin (100 μg/mL) and penicillin (100 U/mL). Cells were transfected with siRNAs or negative control using Lipofectamine RNAiMAX reagent (Invitrogen). The sequences of siRNAs or shRNA were as shown in supplementary Table S1. For CCR2 blockade, cells were treated with 10 μmol/L antagonist RS504393 (MedChemExpress, MCE, HY‐15418) or vehicle control dimethyl sulfoxide (DMSO).

### 2.3 Isolation and identification of exosomes

Cell‐derived exosomes were isolated using Exosome Purification Kit (Norgen Biotek, Canada) according to the instructions. The vesicles were identified by electron microscopic examination and exosome markers (CD63, CD81 and CD9). The concentrations of exosomes were determined by Zetaview (Particle Metrix, Germany).

### 2.4 RNA isolation and quantitative reverse‐transcription polymerase chain reaction (qRT‐PCR)

Total cells or tissues RNAs were extracted by TRIzol (Invitrogen, Carlsbad, CA). QPCR assay was performed with QuantiTect SYBR Green PCR Kit (Takara Bio Inc., Otsu, Japan) on Stepone Real‐Time PCR System (Applied Biosystems, Carlsbad, CA). The primers of lncRNAs were designed by Primer Premier 5.0. The primers were synthesized from Sangon Biotech (Shanghai, China) (Table S2). The relative RNA expression was normalized to GAPDH, which was calculated by the 2^−ΔΔCt^ method (Biorad CFX manager software 3.1).

### 2.5 Macrophages and supernatant preparation

We used 100 ng/mL phorbol myristate acetate (PMA, Sigma, Carlsbad, CA, USA) to induce THP‐1 cells into M0 macrophages. M0 macrophages were incubated with different sources of exosomes (30 μg/mL) for 24 h. The supernatant of co‐incubation was collected by centrifuging at 10,000 g for 5 min and stored at −20°C for later use.

### 2.6 Western blot

Total protein from cells was extracted with RIPA Lysis Buffer (RIPA; Beyotime, Shanghai, China). The whole protein was quantified via bicinchoninic acid (BCA) (Pierce, Waltham, MA, USA) way. A certain amount of protein was loaded for electrophoresis, and the protein after electrophoresis was shifted on a polyvinylidene difluoride (PVDF) membrane (Millipore, Billerica, MA, USA). The PVDF membranes were soaked in 5% skim milk for about 2 h, and then, the membranes were immersed in primary antibodies at 4°C for 18 h and secondary antibodies for 2.5 h. All protein bands were exposed by enhanced chemiluminescence (ECL). Protein expression on the bands was calculated by Image J Software (NIH, Bethesda, MD, USA).

### 2.7 Transwell and transwell‐matrigel assay

Cell migration and invasion assays were performed by transwell insert chambers with 8‐μm pore membranes (Corning Inc., Corning, NY, USA). Approximately 2.8 × 10^5^ cells without FBS were in the upper chamber. The culture solution with 10% FBS existed in the lower chamber. We cultured cells for 24 h. We got rid of the cells on the upper surface of the upper chamber. Then, the chamber surface was washed using phosphate buffer. Ovarian cancer cells in the chamber were soaked into 4% paraformaldehyde. After the chambers were dry, we dyed the cells with 1% crystal violet for about 1.5 min. Migration cells were on the lower surface of the chamber. Migration cells were included at 200 magnifications in nine fields of view. We applied Matrigel Membrane Matrix (40 μg/15 μL; Vigorous Biotechnology Beijing Co., Ltd., Beijing, China) on Boyden chambers with filter inserts (pore size, 8‐μm) to perform cell invasion assays. About 2.8 × 10^5^ cells were plated in the upper chamber. The 650 μL medium with 10% FBS was in the lower chamber for 48 h. Then, cells on the up and lower side of chambers were treated using the method above.

### 2.8 Enzyme‐linked immunosorbent assay (ELISA)

CCL13 human ELISA kit (EHCCL13) was purchased from Invitrogen (ThermoFisher scientific). The protein level in the cell culture supernatant was detected according to the manufacturer's protocol.

### 2.9 Construction of stable knockdown cell lines

For construction of knockdown stable cell lines, shRNAs were inserted into pLKO.1‐TRC plasmid, according to the Addgene instructions. After lentivirus package and infection, cells were selected with 1 μg/mL puromycin. Knockdown efficiency in the stable cell lines was verified by western blot using anti‐CCR2 antibody (Abcam, ab313463).

### 2.10 Xenograft mice model

Animal experiments were performed by the Institutional Animal Care and Use Committee of Beijing Viewsolid Biotechnology Co. Ltd. 2 × 10^6^ wild SKOV3 cells or SKOV3 cells stably expressing CCR2 shRNA or scrambled shRNA in 200 μL PBS were injected subcutaneously into the flank region of Balb/c nude mice (female, six‐week‐old). The mice were randomly divided into groups and injected intravenously with 100 μL different cell co‐incubation supernatant medium or CCL13 recombinant protein solution (50 μg/kg) every 3 days. After 4 weeks, tumours were excised and measured. Tumour volume was calculated using this formula: tumour volume = 0.5 × width^2^ × length.

### 2.11 Statistical analysis

Graphs were constructed by using GraphPad Prism software (version 8.0). All data are presented as mean ± standard deviation. Differences between two groups were compared using the chi‐square test and *t*‐test. Two‐sided *p* < 0.05 was considered statistically significant (*p < 0.05; **p < 0.01; ***p < 0.001).

## 3 RESULTS

### 3.1 SNHG17 is upregulated in ovarian cancer and oestrogen‐induced ovarian cancer‐derived exosomes

**FIGURE 1:**
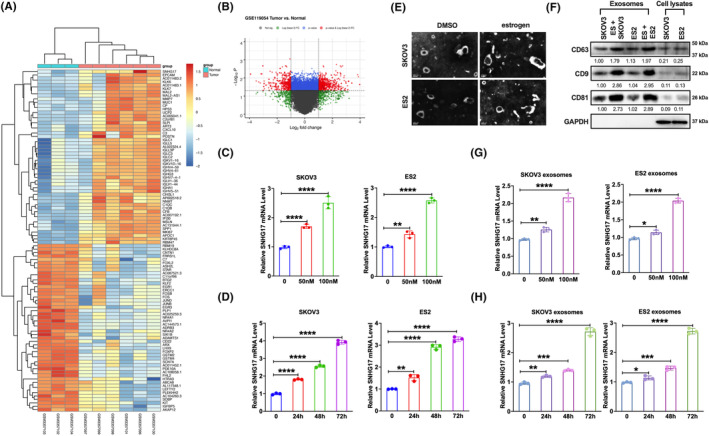
SNHG17 was identified to be increased in ovarian cancer and oestrogen‐treated ovarian cancer cell exosomes. (A) Heatmaps showed the expression of top 50 different expressed genes (DEGs) between tumour and normal tissues of ovarian cancer. (B) Volcano plots analysis between normal and tumour samples in GSE119054. X‐axis and y‐axis illustrated log2 fold change and −log 10 (*p*‐value) for each gene, and the significance cut off (*p*‐value = 0.05). The red, green and grey dots represented up‐regulated, downregulated and non‐significant genes. (C) qPCR analysis of SNHG17 in SKOV3 and ES2 cells with oestrogen treatment at a concentration of 50 nM and 100 nM. (D) qPCR analysis of SNHG17 in SKOV3 and ES2 cells with oestrogen treatment at 0, 24, 48 and 72 h. (E) The representative electron microscopy image of the exosomes derived from ovarian cancer cells SKOV3 and ES2 cells with or without oestrogen treatment. (F) Western blot analysis of exosome markers CD63, CD9 and CD81 in exosomes and cell lysates of SKOV3 and ES2 with or without oestrogen treatment. (G) qPCR analysis of SNHG17 in exosomes derived from SKOV3 and ES2 cells with oestrogen treatment at a concentration of 50 nM and 100 nM. (H) qPCR analysis of SNHG17 in exosomes derived from SKOV3 and ES2 cells with oestrogen treatment at 0, 24, 48 and 72 h. *p < 0.05, **p < 0.01, ***p < 0.001, ****p < 0.0001.

Analysis of GSE119054 revealed that SNHG17 was highly expressed in ovarian cancer tumour group compared to normal control (*p* < 0.001; Figure 1A,B). Since increasing studies reported that oestrogen plays an important role in ovarian cancer,^4^, ^32^, ^33^ we found that the expression of SNHG17 was also increased with the increasing duration and concentration of oestrogen treatment in ovarian cancer cell lines SKOV3 and ES2 (Figure 1C,D). Additionally, we also observed that exosome secretion by SKOV3 and ES2 were increased with oestrogen treatment, as the expression of exosomal markers CD63, CD9 and CD81 was elevated (Figure 1E,F). Furthermore, SNHG17 was also found to be abundantly expressed in the SKOV3 and ES2‐derived exosomes with oestrogen treatment (Figure 1G,H). These results suggest that oestrogen treatment could promote the expression of ovarian cancer‐derived exosome lncRNA SNHG17.

### 3.2 Oestrogen‐induced increase of exosomal lncRNA SNHG17 promotes macrophage M2 polarization

**FIGURE 2:**
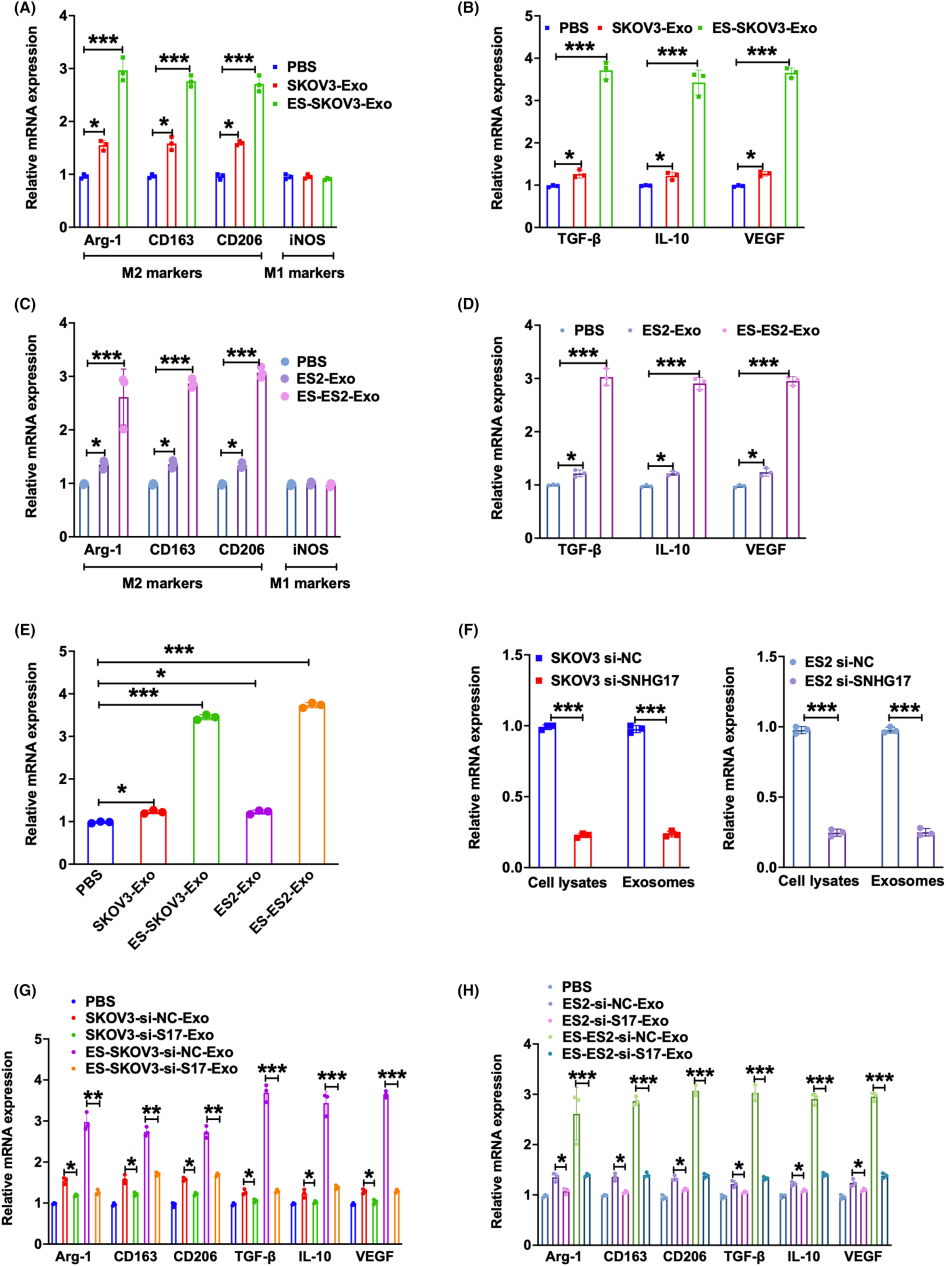
Oestrogen‐treated SKOV3 and ES2 derived exosomes SNHG17 promoted macrophage M2 polarization. THP‐1 cells were treated with 100 ng/mL PMA to achieve the M0 macrophages. Exosomes were obtained from SKOV3 or ES2 cells with or without 50 nM oestrogen treatment. (A–D) qPCR analysis of M2 macrophage biomarkers Arg‐1, CD163, CD206, M1 macrophage biomarker iNOS, M2 macrophage cytokines TGF‐β, IL‐10 and VEGF was measured in macrophages co‐incubated with the above obtained exosomes (30 μg/mL) for 24 h. (E) qPCR analysis of SNHG17 in SKOV3 and ES2 cells or their derived exosomes with or without oestrogen treatment. (F) SKOV3 and ES2 cells were transfected with si‐SNHG17 or si‐NC for 48 h. SNHG17 mRNA level was detected in cell lysates and exosomes. (G,H) SKOV3 and ES2 cells were transfected with si‐SNHG17 or si‐NC for 48 h. The exosomes were extracted from the transfected cells with or without 50 nM oestrogen treatment for 48 h. qPCR analysis of Arg‐1, CD163, CD206, TGF‐β, IL‐10 and VEGF was detected in macrophages co‐incubated with the above obtained exosomes (30 μg/mL) for 24 h. *p < 0.05, **p < 0.01, ***p < 0.001.

To address the potential function of oestrogen‐induced increase of exosomal lncRNA SNHG17 in ovarian cancer, we used 100 ng/mL phorbol 12‐myristate 13‐acetate (PMA)‐induced differentiation of THP‐1 monocyte cells to M0 macrophages as a model to analyse the role of exosomal lncRNA SNHG17 in macrophage polarization. We treated M0 macrophages with different sources of exosomes. The markers of M2 macrophage including arginase‐1 (Arg1), CD163 and CD206 were significantly increased whereas the M1 marker iNOS was almost no change in the group of exosomes with oestrogen treatment compared to control (exosomes without oestrogen treatment), as well as the increased expression of M2‐released cytokines such as transforming growth factor (TGF‐β), interleukin‐10 (IL‐10) and vascular endothelial growth factor (VEGF) in SKOV3 and ES2, respectively (Figure 2A–D). SNHG17 was obviously increased in SKOV3 and ES2 with oestrogen treatment (Figure 2E). To further assess the role of SNHG17 in M2 polarization, we knocked down SNHG17 in SKOV3 and ES2 by transfecting SNHG17‐small interfering RNA (siRNA). SNHG17 was decreased in both cell lysates and exosomes (Figure 2F). We observed that SNHG17 knockdown could reduce the M2 polarization induced by oestrogen compared to control (si‐NC), as the decreased expression of Arg‐1, CD163, CD206, TGF‐β, IL‐10 and VEGF (Figure 2G,H). These results indicate that oestrogen may accelerate the M2 polarization of macrophages by promoting SKOV3 and ES2 cells' exosomal SNHG17 release.

### 3.3 Oestrogen‐induced exosomal SNHG17 modulates macrophage M2 polarization via PI3K/AKT pathway

**FIGURE 3:**
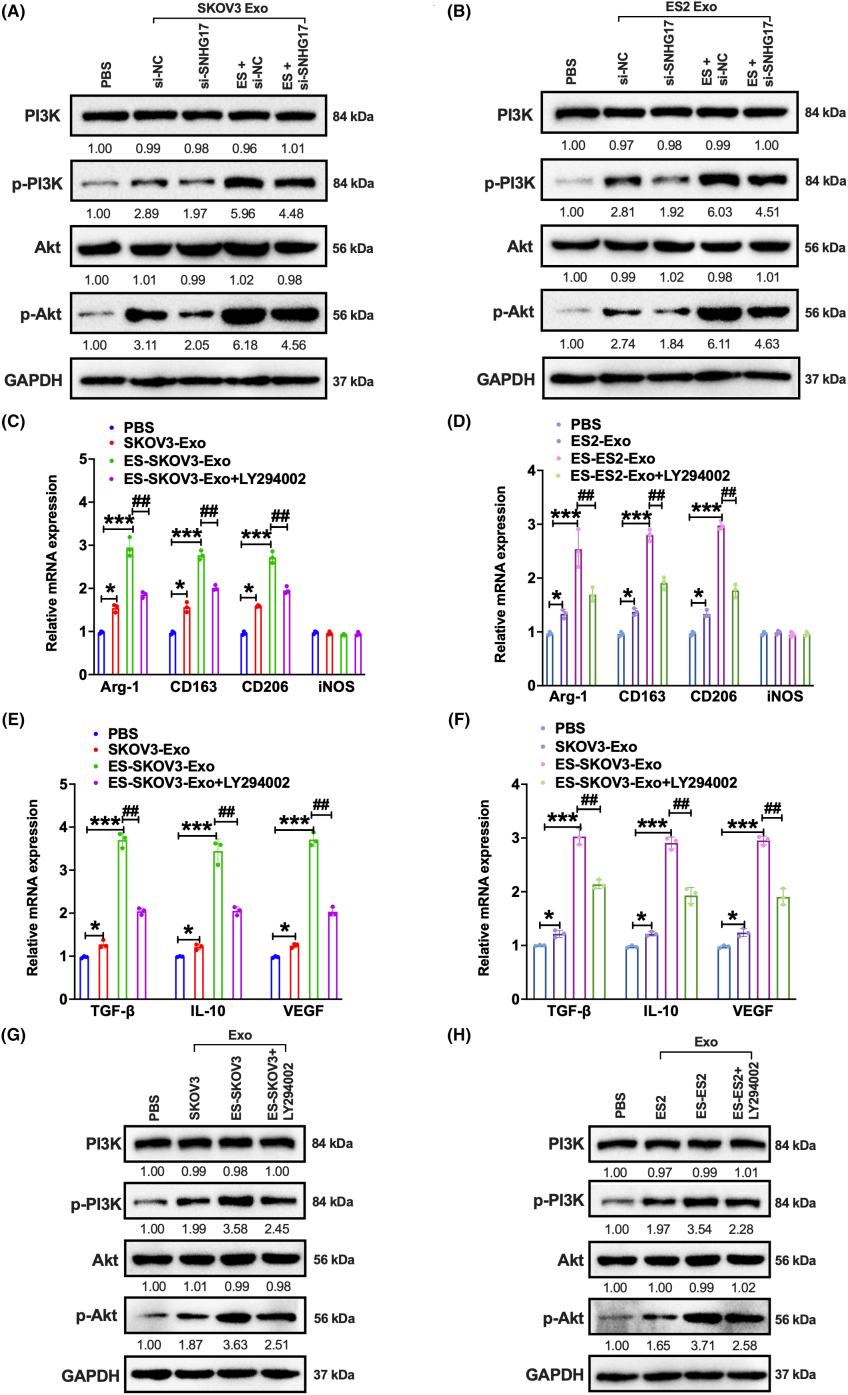
Oestrogen‐induced exosomal SNHG17 promotes macrophage M2 polarization via the PI3K/Akt signalling pathway. (A,B) SKOV3 and ES2 cells were transfected with si‐SNHG17 or si‐NC for 48 h and then treated with 50 nM oestrogen for another 48 h. The above extracted exosomes (30 μg/mL) were added to the macrophages for another 24 h. Western blot analysis of PI3K, p‐PI3K, Akt and p‐Akt was performed. (C–F) Macrophages were treated with four different ways. One group was incubated with PBS. The second group was incubated with exosomes from SKOV3 or ES2 cells without oestrogen treatment. The third group was incubated with exosomes from SKOV3 or ES2 cells with oestrogen treatment. The fourth was treated with LY294002 (12 μM) and incubated with exosomes from SKOV3 or ES2 cells with oestrogen treatment. qPCR analysis of Arg‐1, CD163, CD206, TGF‐β, IL‐10 and VEGF was performed in the above four groups of macrophages with different treatment. *p ＜ 0.05, **p ＜ 0.01, ***p ＜ 0.001 compared to PBS group; ^##^ *p* ＜ 0.01. (G,H) The protein level of PI3K, p‐PI3K, Akt and p‐Akt was detected in the above four groups of macrophages with different treatment.

To further investigate the mechanism by which oestrogen‐induced exosomal SNHG17 modulate macrophage M2 polarization, we examined the PI3K/AKT signalling pathway, which has been reported to be involved in the regulation of M2 polarization.^34^ We observed that exosomes from SKOV3 and ES2 cells with oestrogen treatment could enhance the phosphorylation of PI3K and Akt in macrophages compared to the exosomes from non‐oestrogen‐treated cells (Figure 3A,B). However, knockdown of SNHG17 suppressed this process (Figure 3A,B). Preliminary results suggest that oestrogen treatment may promote M2 polarization via the PI3K/Akt signalling pathway by increasing exosomal SNHG17 secretion. Furthermore, we verified the hypothesis by using PI3K/AKT pathway inhibitor LY294002 in macrophages. The promotion of M2 polarization (high expression of Arg‐1, CD163, CD206, TGF‐β, IL‐10 and VEGF) resulted from oestrogen treatment was significantly inhibited by LY294002 treatment (Figure 3C–F). Consistently, western blot showed that the increased phosphorylated level of PI3K and Akt induced by oestrogen could be suppressed with LY294002 treatment (Figure 3G,H). These results reveal that oestrogen‐induced exosomal SNHG17 could promote macrophage M2 polarization via PI3K/Akt pathway.

### 3.4 Oestrogen‐increased exosomal SNHG17 facilitates ovarian cancer tumour behaviour via M2 polarization in vitro

**FIGURE 4:**
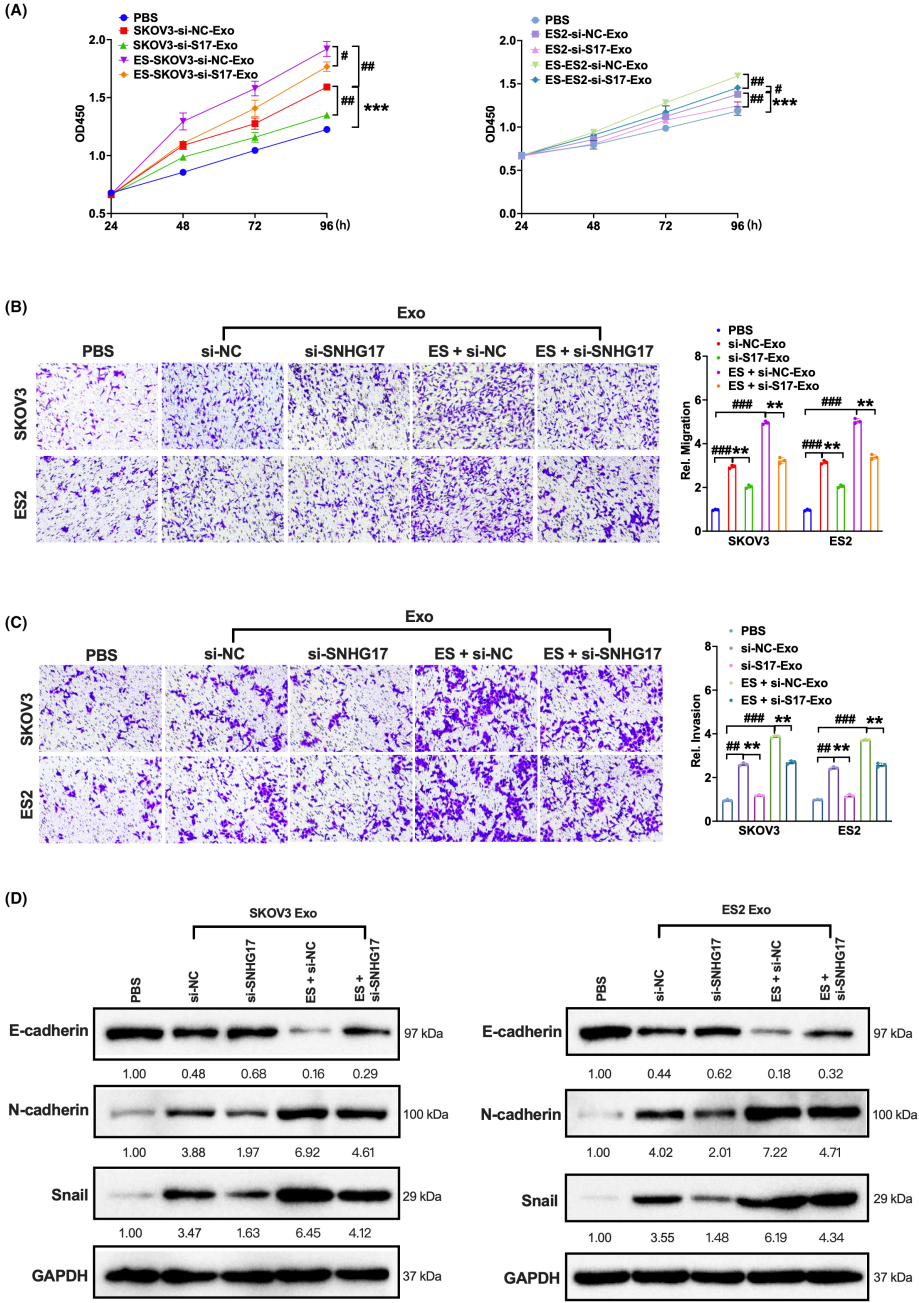
The macrophages incubated with oestrogen‐induced exosomes promote ovarian cancer cell proliferation, migration, invasion and EMT in vitro. SKOV3 and ES2 cells were transfected with si‐SNHG17 or si‐NC for 48 h and then treated with or without 50 nM oestrogen for another 48 h. The above extracted exosomes (30 μg/mL) were added to the macrophages for another 24 h. The obtained supernatant was used to treat SKOV3 or ES2 cells. (A) CCK‐8 analysis was performed to examine cell proliferation. ^#^ *p* ＜ 0.05, ^##^ *p* ＜ 0.01; ***p ＜ 0.001 compared to PBS group. (B,C) Transwell and transwell‐matrigel assays were performed to examine cell migration and invasion. ^##^ *p* ＜ 0.01, ^###^ *p* ＜ 0.001 compared to PBS group; **p ＜ 0.01. (D) The EMT‐related proteins E‐cadherin, N‐cadherin and Snail were detected. *p ＜ 0.05, **p ＜ 0.01, ***p ＜ 0.001.

Next, we explored the biological effect of the M2 polarization triggered by oestrogen‐increased exosomal SNHG17. We collected the supernatant of the co‐culture of macrophages and exosomes with different treatment; then, we added the above supernatant in SKOV3 and ES2 cells. We performed CCK‐8, transwell and transwell‐matrigel assays. The results showed that the increased cell viability, cell migratory and invasive abilities caused by the culture of supernatant from the co‐culture of macrophage and exosomes with oestrogen treatment could be blocked by the supernatant from the co‐culture of macrophage and SNHG17‐knockdown exosomes with oestrogen treatment (Figure 4A–C). The western blot analysis of epithelial‐mesenchymal transition (EMT)‐related proteins was consistent with the above results, as the increased N‐cadherin and Snail expression and decreased E‐cadherin expression induced by the supernatant from the co‐culture of macrophage and exosomes with oestrogen treatment were blocked by the supernatant from the co‐culture of macrophage and SNHG17‐knockdown exosomes with oestrogen treatment (Figure 4D). These data suggest that oestrogen‐increased exosomal SNHG17 could promote ovarian cancer tumour behaviour including proliferation, migration, invasion and EMT via M2 polarization in vitro.

### 3.5 Oestrogen‐increased exosomal SNHG17 promotes tumour growth and metastasis in the xenograft nude mouse model

**FIGURE 5:**
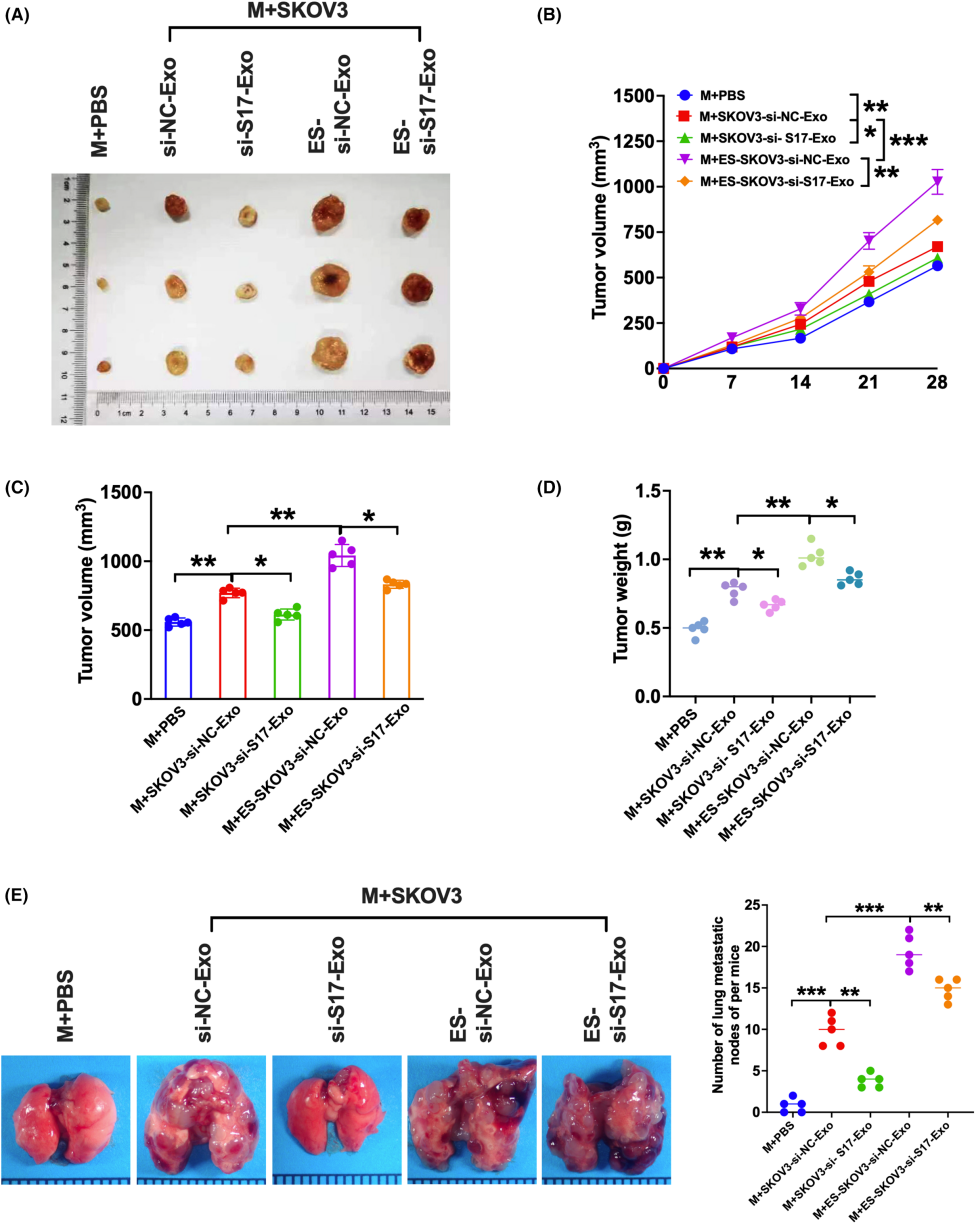
Oestrogen‐induced exosomal SNHG17 promotes ovarian cancer tumour growth and metastasis via macrophage M2 polarization in vivo. SKOV3 cells were transfected with si‐SNHG17 or si‐NC for 48 h and then treated with or without 50 nM oestrogen for another 48 h. The above extracted exosomes (30 μg/mL) were added to the macrophages for another 24 h. The obtained supernatants were used to inject into the xenograft mice. (A) Representative image of xenograft tumours from different groups. (B) The tumour volume was measured and calculated at the indicated time points. (C) The tumour volume at Day 28 was recorded as the column chart. (D) The tumours were obtained after mice were sacrificed and weighed at Day 28. (E) The morphological change of the lungs was observed in different groups as shown in the left. The metastatic nodes in the lungs were calculated in different groups as shown in the right (*n* = 5). *p < 0.05, **p < 0.01, ***p < 0.001.

Furthermore, we confirmed the supernatant effect on tumour growth and metastasis in vivo using the murine tumour xenograft model. Compared to the controls, the larger and heavier tumours caused by the supernatant from macrophages with the exosome from SKOV3 were much more significantly increased additively with oestrogen treatment (Figure 5A–D). Meanwhile, the tumour growth promoting effects were reduced by exosomes with SNHG17‐knockdown (Figure 5A–D). Moreover, the severe pulmonary metastasis induced by oestrogen treatment was also significantly abrogated by SNHG17‐knockdown (Figure 5E). These results suggest that oestrogen‐increased exosomal SNHG17 promotes tumour growth and metastasis via macrophage M2 polarization in vivo.

### 3.6 Oestrogen‐induced M2 polarization by promoting the release of exosomal SNHG17 promotes proliferation, migration and invasion of ovarian cancer by releasing CCL13

**FIGURE 6:**
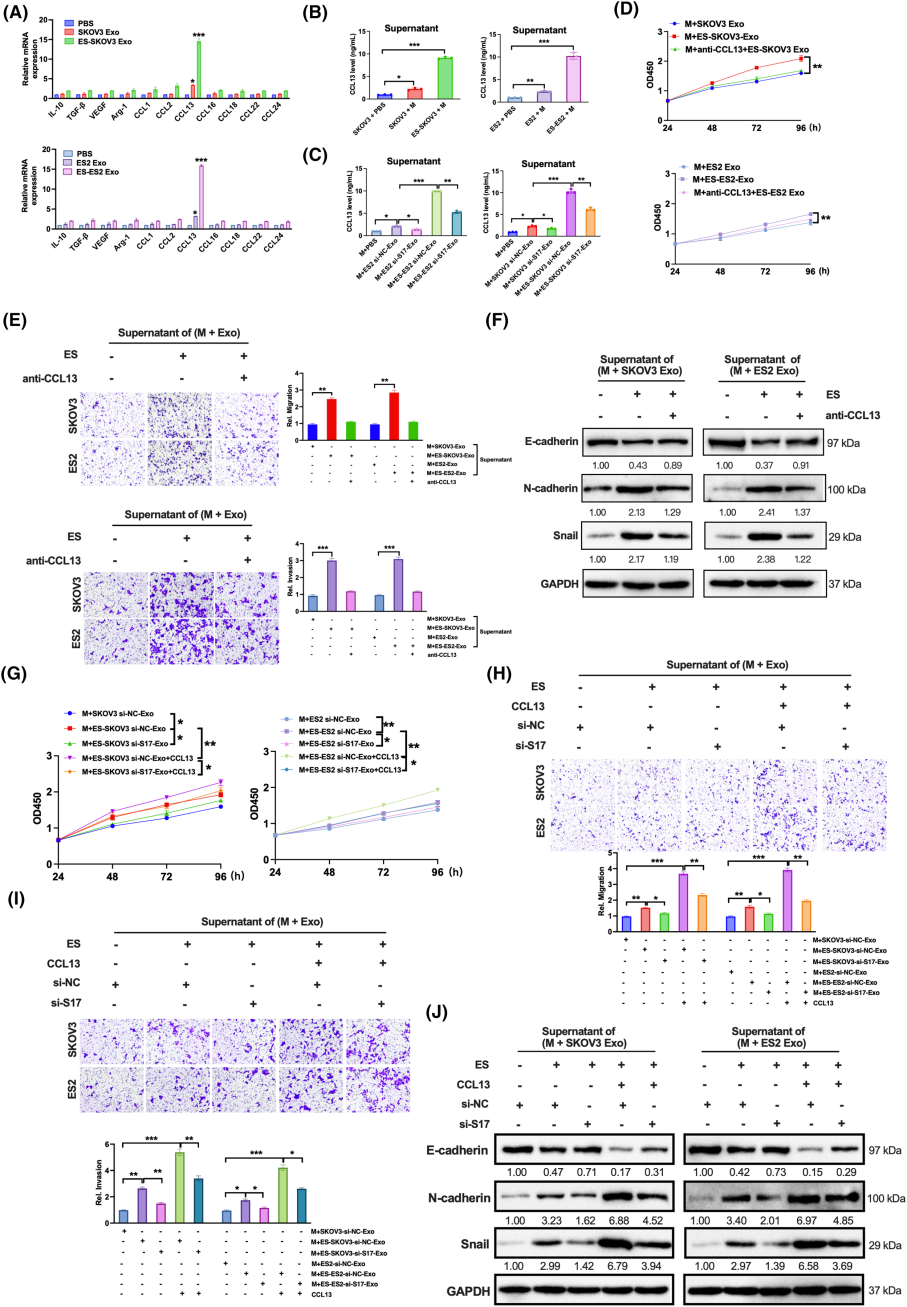
Oestrogen‐induced exosomal SNHG17 facilitates ovarian cancer cell proliferation, migration, invasion and EMT by promoting M2 macrophage releasing CCL13. (A) Macrophages were incubated with PBS or exosomes from SKOV3 or ES2 cells with or without oestrogen treatment. The relative mRNA level of macrophage cytokines was performed by RT‐PCR. (B) CCL13 content in the supernatant of macrophages was measured by ELISA. (C) SKOV3 or ES2 cells were transfected with si‐SNHG17 or si‐NC for 48 h and then treated with or without oestrogen treatment for 24 h. CCL13 content was measured in the supernatant from macrophages incubated with exosomes. (D) Macrophages were incubated with exosomes from SKOV3 or ES2 cells with or without oestrogen treatment, and/or with anti‐CCL13 antibody (1.5 μg/mL). Cell proliferation of SKOV3 and ES2 treated with the above supernatants was analysed by CCK‐8. (E) Cell migration and cell invasion were analysed. (F) EMT‐related proteins were measured. (G) SKOV3 or ES2 cells were transfected with si‐SNHG17 or si‐NC for 48 h and then treated with or without oestrogen treatment for 24 h, and/or with CCL13 recombination protein (30 ng/mL). The supernatants were obtained from the macrophages incubated with the exosomes from above groups. SKOV3 or ES2 were treated with the above supernatants. Cell proliferation (G), cell migration (H), cell invasion (I) and EMT‐related proteins were analysed. *p < 0.05, **p < 0.01, ***p < 0.001.

Based on the above findings, we hypothesized that factors secreted by macrophages co‐cultured with exosomes with oestrogen treatment could promote ovarian cancer tumour growth and metastasis in vitro and in vivo. To identify the factor, we tested the macrophage‐related cytokines including IL‐10, TGF‐β, VEGF, Arg‐1, CCL1, CCL2, CCL13, CCL16, CCL18, CCL22 and CCL24. As shown in Figure 6A,B, CCL13 was verified and validated to be upregulated in supernatant of the macrophages incubated with exosomes with oestrogen treatment in both SKOV3 and ES2 cells. What's more, the release of CCL13 was reduced when the supernatant was from the macrophages incubated with SNHG17‐knockdown exosomes with oestrogen treatment (Figure 6C). Then, we investigated the biological effects of CCL13 on cell proliferation, migration, invasion and EMT. The strengthening effect on tumour behaviour induced by oestrogen treatment could be blocked when co‐treatment with CCL13 neutralizing antibody (anti‐CCL13, Figure 6D–F). Next, we assessed whether oestrogen‐induced exosomal SNHG17 could promote the effects via CCL13. The increased proliferation, migration, invasion and EMT caused by the supernatant of co‐treatment with oestrogen and CCL13 could be significantly reduced by the supernatant of co‐incubation with SNHG17 knockdown (Figure 6G–J). These results show that oestrogen‐induced exosomal SNHG17 has the facilitating effect on ovarian cancer by promoting M2 releasing CCL13.

### 3.7 The CCL13‐CCR2‐M2 macrophage axis is involved in oestrogen‐induced ovarian cancer tumour growth

**FIGURE 7:**
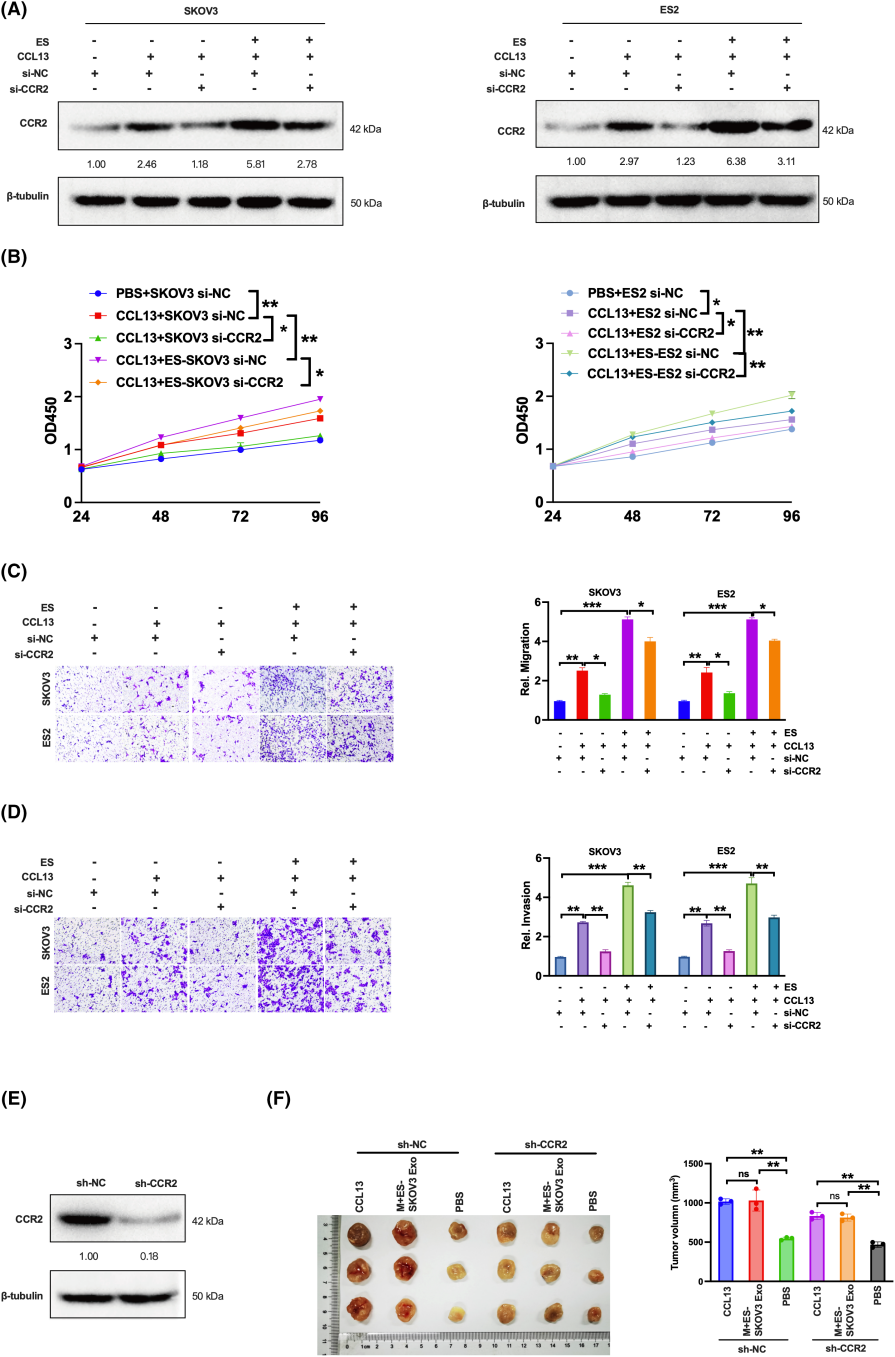
CCL13‐CCR2 axis is involved in oestrogen‐induced ovarian cancer tumour behaviours in vitro and in vivo. SKOV3 or ES2 cells were transfected with si‐CCR2 or si‐NC for 48 h and then treated with or without oestrogen treatment for 24 h, and/or with CCL13 recombination protein (30 ng/mL). (A) The protein level of CCR2 was detected by western blot. Cell proliferation (B), cell migration (C) and cell invasion (D) were analysed. (E) The protein level of CCR2 in SKOV3 cells transfected with sh‐NC or sh‐CCR2 was examined by western blot. (F) Mice were inoculated with SKOV3 cells transfected with sh‐NC or sh‐CCR2 and then injected with or without CCL13 recombination protein (45 μg/kg), or the supernatants from macrophages incubated with oestrogen‐treated SKOV3 cell exosomes, or PBS. The tumour volume was measured and calculated at Day 28. *p < 0.05, **p < 0.01, ***p < 0.001.

It has been known that CCR2 is the chemokine receptor of CCL13.^35^, ^36^, ^37^ To clarify whether CCL13–CCR2 was involved in the oestrogen‐induced exosomal SNHG17 on ovarian cancer tumour growth, we knocked down CCR2 in SKOV3 and ES2 cells and added CCL13 recombination protein. Firstly, we observed that CCR2 expression was elevated in CCL13‐treated group compared with the control group, and oestrogen treatment cells showed a more substantial facilitation effect. Whereas the CCR2 level was significantly decreased in si‐CCR2‐transfected cells compared to si‐NC‐transfected cells treated with CCL13 (Figure 7A), the CCR2 expression was also obviously downregulated in CCR2‐knockdown cells compared to control group treated with oestrogen (Figure 7A). Next, we explored whether CCR2 was involved in the enhanced effect of CCL13 on ovarian cancer cell growth in vitro. As shown in Figure 7B–D, the increased proliferation, migration and invasion caused by CCL13 treatment could be reduced by CCR2 knockdown both in SKOV3 and ES2 cells (Figure 7B–D), and CCR2 knockdown could also eliminated the oestrogen‐induced effects. Furthermore, we constructed stable CCR2‐knockdown SKOV3 cell lines and confirmed the silence of CCR2 expression in established cell lines (Figure 7E). In the xenograft mouse model, the supernatant from the co‐incubation of macrophages and oestrogen‐treated SKOV3 exosomes could promote tumour growth, as well as CCL13 treatment, which were suppressed by CCR2 knockdown (Figure 7F). These results suggest that oestrogen‐induced exosomal SNHG17 could promote ovarian cancer tumour growth via macrophage M2 polarization, and that CCL13‐CCR2 was responsible for these effects.

To further confirm that CCR2 was involved in oestrogen‐induced exosomes on ovarian cancer tumour growth, we introduced RS504393, a CCR2 antagonist.^38^ When SKOV3 or ES2 cells were treated with RS504393, the protein level of CCR2 was obviously decreased than the corresponding control (Figure S1A). Moreover, downregulation of CCR2 could weaken the promoting role of CCL13 on cell proliferation, cell migration and cell invasion (Figure S1B–D), and CCR2 downregulation could also partially reduce the oestrogen‐induced effects (Figure S1B–D). Thus, we concluded that CCR2 played a vital role in the accelerative effects of oestrogen‐induced exosomal SNHG17 on ovarian cancer tumour growth in vitro.

## 4 DISCUSSION

Oestrogen is one of the risk factors for ovarian cancer development, progression and metastasis.^39^, ^40^ However, the underlying molecular mechanisms remain elusive. Therefore, elucidating the molecular mechanism underlying oestrogen‐induced ovarian cancer tumorigenesis is making sense for the development of potential therapeutic strategies for improving the survival of patients with oestrogen‐related ovarian cancer. In this study, we revealed that oestrogen‐induced exosomal lncRNA SNHG17 facilitated tumour growth and metastasis in vitro and in vivo. Oestrogen‐induced exosomal lncRNA SNHG17 could promote macrophage M2 polarization via PI3K/Akt signalling pathway. Furthermore, we also identified that exosomal lncRNA SNHG17 promoted ovarian cancer via CCL13‐CCR2‐M2 macrophage axis.

Increasing studies showed that lncRNAs are important regulators of tumorigenesis and development in various cancers.^41^, ^42^, ^43^ In the present study, we discovered that SNHG17 was upregulated in ovarian cancer. Upon oestrogen treatment, the exosomes derived from SKOV3 and ES2 cells and the exosome‐delivered SNHG17 were significantly increased. These results indicate that exosomal SNHG17 may act as ‘oncogene’ in ovarian cancer. It was reported that SNHG17 silence could inhibit the tumorigenesis of epithelial ovarian cancer via the miR‐485‐5p/AKT1 axis.^44^ Besides, SNHG17 was also found to play oncogenic roles in prostate cancer,^15^ rectal cancer,^17^ lung adenocarcinoma^18^ and ovarian cancer.^19^, ^20^ Consistently, our results confirm the oncogenic role of SNHG17 in ovarian cancer, that is exosomal SNHG17 could promote ovarian cancer cell proliferation, migration, invasion and EMT in vitro and tumour growth and metastasis in vivo.

The vital roles of TAMs, as one of key components of TME, have been studied in the regulations of tumour progression, development and metastasis. Multiple research projects showed that tumour‐derived exosomes could promote tumours development by regulating the M1/M2 polarization of TAMs.^45^, ^46^, ^47^ It was reported that exosomal microRNAs could induce tumour‐associated macrophages via PPARγ during tumour progression in SHH medulloblastoma.^48^ It was studied that loss of XIST could activate MSN‐c‐Met via exosomal miRNA to promote brain metastasis in breast cancer.^49^ Exosomal miR‐181a‐5p derived from SAOS‐2 cells was found to promote macrophage M2 polarization by targeting RORA.^50^ However, there is still little research on the function of ovarian cancer‐derived exosomes in TAM polarization and tumour development. In this study, we discovered that exosomes from SKOV3 and ES2 cells could promote the M2 polarization of macrophages. Moreover, with oestrogen treatment, exosomes could accelerate the effects by increasing exosomal SNHG17 expression. PI3K/Akt signalling pathway has been reported to be involved in the macrophage activation and M1/M2 polarization.^51^ Herein, we observed that ovarian cancer cells secreted exosomes could enhance the M2 polarization of macrophages by activating PI3K/Akt signalling pathway. Oestrogen treatment could aggravate the effect by upregulating exosomal SNHG17 expression.

Accumulating evidence suggests that M2 macrophages could modulate tumour behaviours by secreting factors in tumour microenvironment. For example, M2 phenotype‐macrophages could secrete IL‐4, IL‐5 and IL‐6 to enhance angiogenesis, immunosuppression and matrix remodelling during tumour progression.^52^ In this study, CCL13 was identified to be the factor released by M2 macrophages exposed to oestrogen. In addition, we confirmed that oestrogen‐induced exosomal CCL13 could promote CCL13 release to regulate ovarian cancer cell proliferation, migration, invasion and EMT. Furthermore, we verified that CCR2 was the receptor of CCL13 when ovarian cancer with oestrogen treatment. The CCL13‐CCR2 axis in ovarian cancer tumour growth was investigated in vitro and in vivo. These results indicate that oestrogen‐induced ovarian cancer exosomal SNHG17 could promote tumour growth by regulating M2 polarization via CCL13‐CCR2 axis.

**FIGURE 8:**
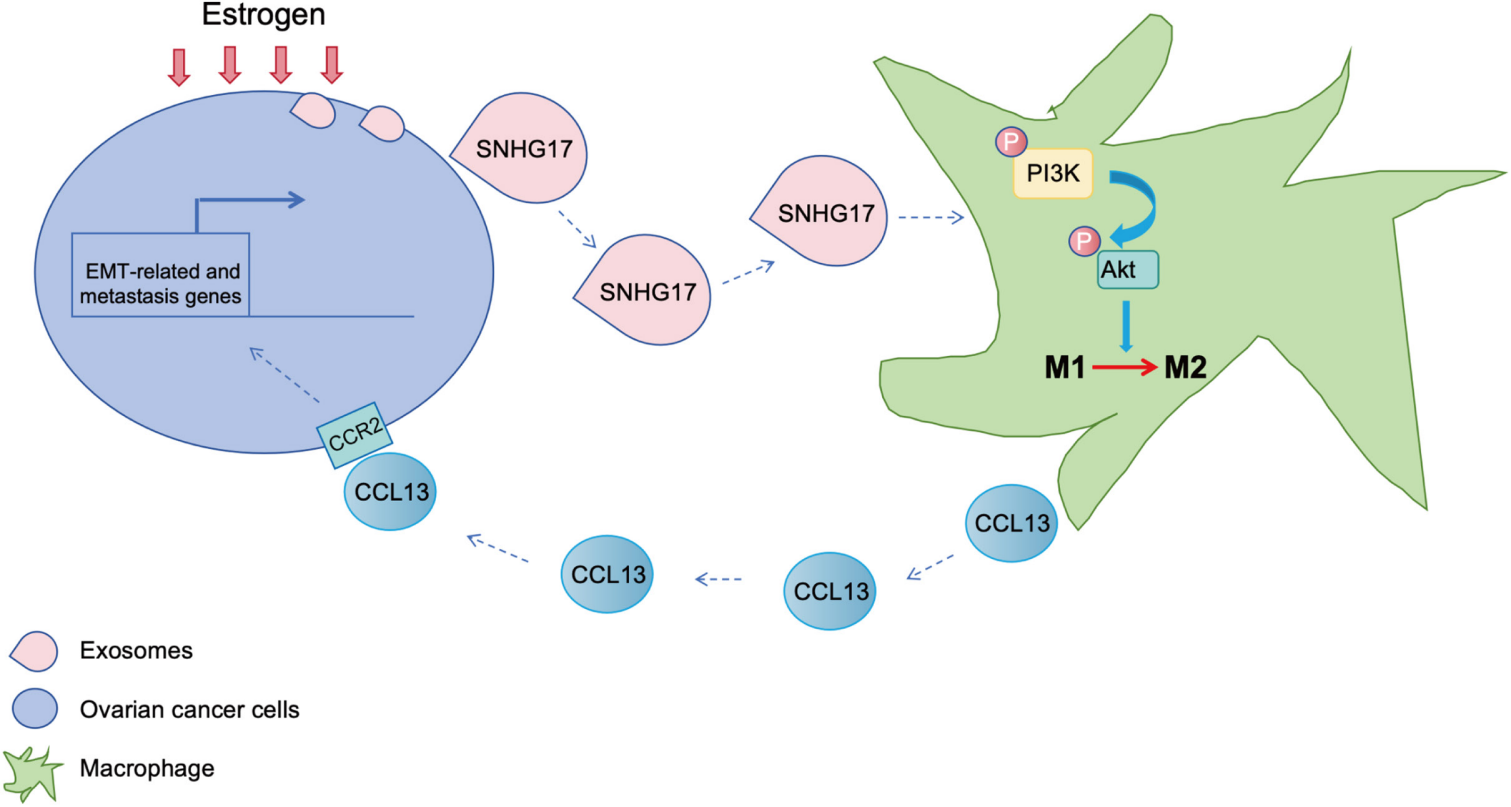
The graphic illustration for role of oestrogen‐induced exosomal SNHG17‐CCL13‐CCR2‐M2 macrophage axis in ovarian cancer. Oestrogen treatment promotes ovarian cancer exosomes SNHG17 secretion, of which facilitates the macrophage M2 polarization via PI3K/Akt pathway. The activated M2 macrophage releases CCL13, binding to CCR2 and thus promotes ovarian cancer tumour growth and metastasis in vitro and in vivo.

To sum up, our work demonstrates that oestrogen treatment promoted ovarian cancer exosomes SNHG17 secretion, of which facilitated the macrophage M2 polarization via PI3K/Akt pathway. The activated M2 macrophage released CCL13, binding to CCR2 and thus promoted ovarian cancer tumour growth and metastasis in vitro and in vivo (Figure 8). Our results provide novel insights of the mechanisms underlying the roles of exosomes lncRNAs in oestrogen‐related ovarian cancer.

## AUTHOR CONTRIBUTIONS

**Haiyan Liang:** Conceptualization (equal); project administration (equal). **Shuo Geng:** Conceptualization (equal); project administration (equal); writing – original draft (equal). **Yadong Wang:** Data curation (equal); investigation (equal). **Qing Fang:** Investigation (supporting); methodology (supporting). **Yongfeng Xin:** Data curation (supporting); formal analysis (supporting). **Yanqing Li:** Investigation (supporting); methodology (supporting).

## CONFLICT OF INTEREST STATEMENT

The authors declare that they have no competing interests.

## Supporting information


**Figure S1.** CCR2 antagonist partially blocks the promoting effects caused by oestrogen induction via the release of CCL13. SKOV3 or ES2 cells were treated with CCR2 antagonist RS504393 (10 μmol/L) for 24 h and then treated with or without oestrogen treatment for 24 h, and/or with CCL13 recombination protein (30 ng/mL). (A) CCR2 expression at protein level was detected by western blot. (B) Cell proliferation (B), cell migration (C), and cell invasion (D) were performed by CCK‐8, transwell and transwell‐matrigel assays. *p < 0.05, **p < 0.01, ***p < 0.001.

**Table S1.** Sequences for siRNAs or shRNA.

**Table S2.** Sequences of qPCR primers.

## Data Availability

The datasets used and/or analysed during the current study are available from the corresponding author on reasonable request.
