# Supplementary figures and images for: Tumour‐derived exosome SNHG17 induced by oestrogen contributes to ovarian cancer progression via the CCL13–CCR2–M2 macrophage axis
Source: J Cell Mol Med. 2024 Apr 28;28(9):e18315. doi: 10.1111/jcmm.18315 (PMC11056704; doi:10.1111/jcmm.18315)

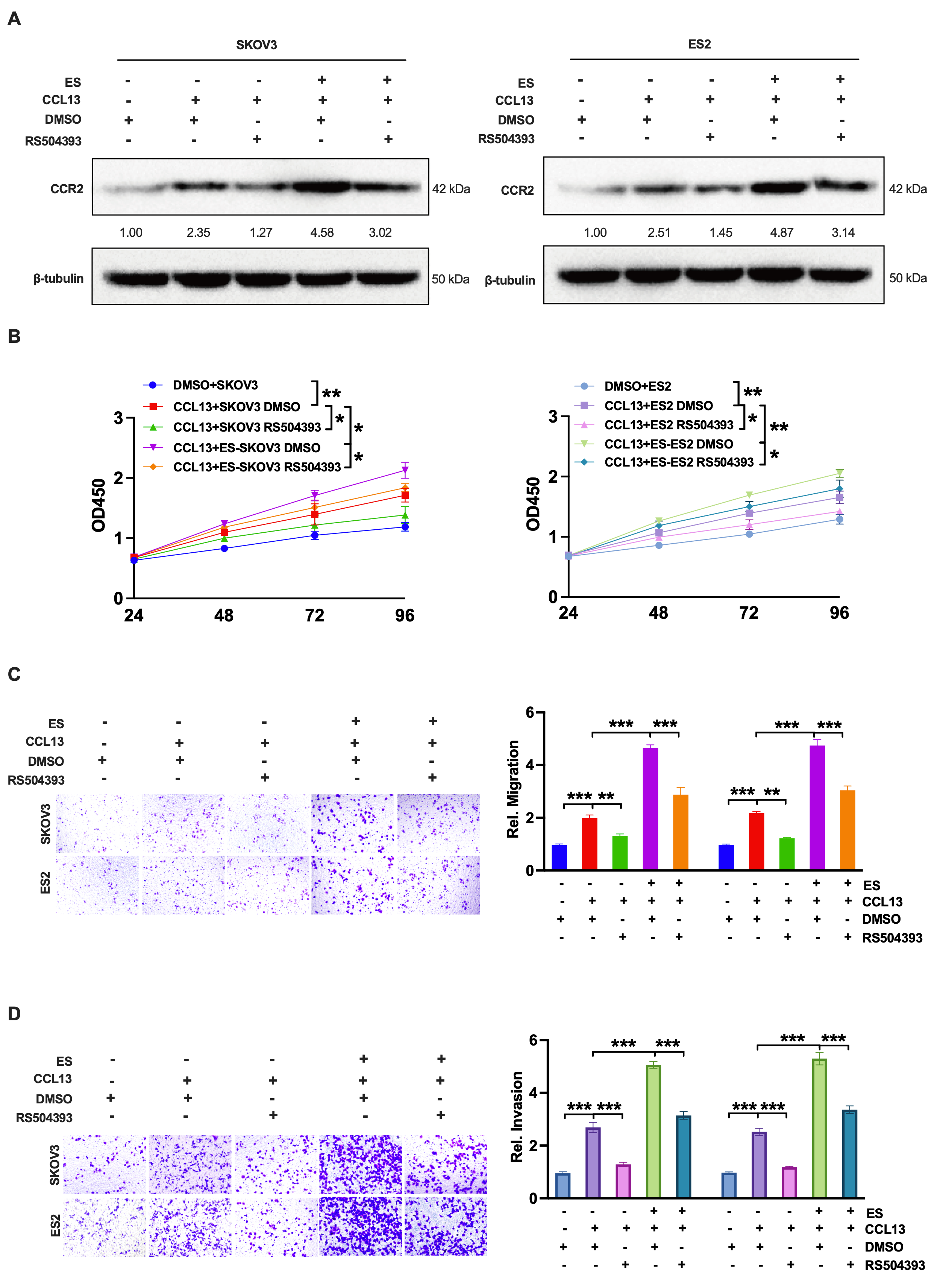

Supplement: Supplementary file 1 — Figure S1. CCR2 antagonist partially blocks the promoting effects caused by oestrogen induction via the release of CCL13. SKOV3 or ES2 cells were treated with CCR2 antagonist RS504393 (10 μmol/L) for 24 h and then treated with or without oestrogen treatment for 24 h, and/or with CCL13 recombination protein (30 ng/mL). (A) CCR2 expression at protein level was detected by western blot. (B) Cell proliferation (B), cell migration (C), and cell invasion (D) were performed by CCK‐8, transwell and transwell‐matrigel assays. *p < 0.05, **p < 0.01, ***p < 0.001. [file JCMM-28-e18315-s001.tiff]
