# Supplementary material for: Tumour‐derived exosome SNHG17 induced by oestrogen contributes to ovarian cancer progression via the CCL13–CCR2–M2 macrophage axis
Source: J Cell Mol Med. 2024 Apr 28;28(9):e18315. doi: 10.1111/jcmm.18315 (PMC11056704; doi:10.1111/jcmm.18315)
Supplement: Supplementary file 2 — Table S1. Sequences for siRNAs or shRNA. [file JCMM-28-e18315-s003.docx]

**Supplementary Table 1. Sequences for siRNAs or shRNA**

| **siRNAs** | **Sequences(5’-3’)** |
| --- | --- |
| si-SNHG17 Sense | GCACCCCAUCUCUCGAAAUCU |
| si-SNHG17 Antisense | AGAUUUCGAGAGAUGGGGUGC |
| si-NC Sense | GCCCUCUACAAUCCGCACUUA |
| si-NC Antisense | UAAGUGCGGAUUGUAGAGGGC |
| **shRNA** | **Oligos (5’-3’)** |
| sh-SNHG17 | ACCTCGCACCCCATCTCTCGAAATCTTCAAGAGAGATTTCGAGAGATGGGGTGCTT |
| sh-NC | ACCTCGCCCTCTACAATCCGCACTTATCAAGAGTAAGTGCGGATTGTAGAGGGCTT |
