# Supplementary material for: Tumour‐derived exosome SNHG17 induced by oestrogen contributes to ovarian cancer progression via the CCL13–CCR2–M2 macrophage axis
Source: J Cell Mol Med. 2024 Apr 28;28(9):e18315. doi: 10.1111/jcmm.18315 (PMC11056704; doi:10.1111/jcmm.18315)
Supplement: Supplementary file 3 — Table S2. Sequences of qPCR primers. [file JCMM-28-e18315-s002.docx]

**Supplementary Table 2. Sequences of qPCR primers**

| **Gene** | **Sequences (5'-3')** |
| --- | --- |
| SNHG17 Forward | CCTGGGAGTGTCACATGACTG |
| SNHG17 Reverse | ACAACACATCCCATGGAGCC |
| Arg 1 Forward | ACTTAAAGAACAAGAGTGTGATGTG |
| Arg 1 Reverse | TACAGGGAGTCACCCAGGAG |
| CD163 Forward | ACCCAGTGAGTTCAGCCTTT |
| CD163 Reverse | CCAGGTGTGGCTCTGCATTT |
| CD206 Forward | CCAAACGCCTTCATTTGCCA |
| CD206 Reverse | GATCTGACTCCGGGCATCTG |
| iNOS Forward | GCCAGGCCACCTCTATGTTT |
| iNOS Reverse | GAGGCTCCGATCAATCCAGG |
| TGF-β Forward | ACCTGCCACAGATCCCCTAT |
| TGF-β Reverse | CCGGTAGTGAACCCGTTGAT |
| IL-10 Forward | TGCAAAAGAAGGCATGCACA |
| IL-10 Reverse | CGCCTTGATGTCTGGGTCTT |
| VEGF Forward | CTCACCAAGGCCAGCACATA |
| VEGF Reverse | GGCTCCAGGGCATTAGACAG |
| CCL1 Forward | CTTGCTGCTAGCTGGGATGT |
| CCL1 Reverse | CAGGGGAATCTCTTGCTCCG |
| CCL2 Forward | ACCTGGACAAGCAAACCCAA |
| CCL2 Reverse | ATGTAAGCCCCACCCTCTGA |
| CCL13 Forward | ACGTCCCATCTACTTGCTGC |
| CCL13 Reverse | GATGACAGCCTTCTGGGGAC |
| CCL16 Forward | TTCGTCACCAAGAGGAACCG |
| CCL16 Reverse | CACTGGGAGTTGAGGAGCTG |
| CCL18 Forward | CTTGTCCTCGTCTGCACCAT |
| CCL18 Reverse | CTGGGGGCTGGTTTCAGAAT |
| CCL22 Forward | GAGATCTGTGCCGATCCCAG |
| CCL22 Reverse | AGGGCCAGGGGACATCTAAT |
| CCL24 Forward | CTGCAAGGACCCGAGCTATT |
| CCL24 Reverse | GAGCCCGTAGGGATGATGTG |
| GAPDH Forward | CAAGCAGACAGAGAGCCTGG |
| GAPDH Reverse | TGTCTTAGCCAGCCCAGAAC |
